# Regional disparities in immunization services in Ghana through a bottleneck analysis approach: implications for sustaining national gains in immunization

**DOI:** 10.1186/s13690-017-0179-7

**Published:** 2017-03-13

**Authors:** A. E. Yawson, G. Bonsu, L. K. Senaya, A. O. Yawson, J. B. Eleeza, J. K. Awoonor-Williams, H. K. Banskota, E. E. A. Agongo

**Affiliations:** 1 Department of Community Health, School of Public Health, College of Health Sciences, University of Ghana, Room 46, P. O. Box 4236, Korle-Bu, Accra, Ghana; 2 National Expanded Programme on Immunization, Public Health Directorate, Ghana Health Service, Accra, Ghana; 3 New Juabeng Municipal Health Directorate, Ghana Health Service, Koforidua, Ghana; 4 Department of Child Health, Korle-Bu Teaching Hospital, Accra, Ghana; 5 Greater Accra Health Directorate, Ghana Health Service, Accra, Ghana; 6 Office of the Regional Director of Health Services, Ghana Health Services, Upper East Region, Bolgatanga, Ghana; 7 UNICEF Country Office, Accra, Ghana; 8 Office of the Director of Policy, Planning, Monitoring and Evaluation Division, Ghana Health Service, Accra, Ghana

**Keywords:** Immunization, Bottleneck analysis approach, Health service scale up, Ghana

## Abstract

**Background:**

Immunization is considered one of the most cost effective public health interventions for reducing child morbidity, mortality and disability. The aim of this work is to describe the application of the Bottleneck analysis (BNA) process to assess gaps in immunization services in Ghana and implications for sustaining the gains in Immunization coverage.

**Methods:**

A national assessment was conducted in May 2015, through use of desk reviews, field visits and key informant interviews. Quantitative data were analysed with the BNA Tool (an excel-based tool) based directly on service coverage data and programme monitoring and review reports in Ghana. Outputs were generated based on service coverage indicators; supply side/health system factors (commodities, human resource and access), demand side (service utilisation) and quality/effective coverage. National targets were used as benchmarks to assess gaps in coverage indicators.

**Results:**

In all, only 50% of regions and districts had health facilities with at least 80% of health care workers training provided in-service training on routine immunization; only 40% of district had communities with functional fixed or outreach EPI service delivery point and over 70% of regions and districts had challenges with effective coverage of infants aged 0-11 months fully immunized during the past year.

Other key health system bottlenecks included, limited number of fixed and outreach sites, difficult to reach island communities along the Volta Basin, inadequate storage facilities for vaccines at lower levels, stock out of vaccines and auto destruct syringes and absence of updated policies/field guides at services delivery points/facilities. In addition, inadequate in-service training in routine Immunization and absence of good quality data were major challenges. Demand side bottlenecks included fear of mothers on the safety of multiple vaccines and limited active involvement of communities in Immunization service delivery.

**Conclusion:**

The BNA tool and approach provided data driven planning of health service in Ghana. This resulted in the development of regional and national operational plans for immunization and will be the baseline for evaluating the national programme in three years.

## Background

Global attention and efforts have focused on the attainment of the health related MDGs, especially MDGs 4 and improving child survival [1, 2]. Immunization is considered one of the most cost effective public health interventions for reducing child morbidity, mortality and disability. Globally, an estimated 2.5 million child deaths and 600 000 adult deaths are prevented annually through Immunization [3]. The global effort to use vaccination as a public health intervention began when the World Health Organization launched the Expanded Programme on Immunization (EPI) in 1974 [4].

To benefit from the full direct and indirect effects of Immunization, the WHO has come up with the Global Vaccine Action Plan which demands on countries to achieve 90% coverage for all antigens and at least 80% coverage in all antigens in 80% of districts by the year 2020 [2].

In Ghana EPI was introduced in 1978 with six antigens which has increased over the years to twelve in 2013 [5]. The expanded programme on Immunization (EPI) in Ghana is organized along the hierarchical organizational structure of the Ghana Health Delivery System; national, regional, district, sub-district and community levels based on the World Health Organization recommendations. At all levels, static and outreach Immunization services are provided in addition to other child health interventions. Immunization is delivered through routine EPI services and national immunization campaigns for poliomyelitis and measles vaccinations.

Ghana has made great progress in vaccination coverage from 60% in 1988 to 89.9% in 2014 using the third dose of the pentavalent vaccine as a proxy. There is however a challenge on how to sustain the gains made as there is an indication of stagnation of the national vaccination coverage from 2008 to 2014 [6, 7]. In 2014, only 69% of districts achieved 80% and above for the third dose of pentavalent vaccine, which falls short of the 80% target [5].

Barriers to reaching every child with the full complement of vaccines have been identified in some settings to include maternal education, distance to facilities, inadequate vaccines, poorly trained and motivated human resources and poor quality of services. In order to address the stagnation and inequities at the different levels of the health delivery system, it is imperative to identify area specific barriers and bottlenecks affecting reaching every child with live saving vaccines. The Health Summit in 2013 of the Ministry of Health of Ghana recommended application of the Bottleneck Analysis Tool Kit for gap analysis to develop appropriate responses in line with the Ghana EPI Comprehensive Multi-Year plan 2015-2019 [8].

The Bottleneck Analysis (BNA) approach is a conceptual framework for effective planning, implementation and monitoring of interventions. It is based on Tanahashi’s Health Service Coverage Evaluation methodology [9] which examines supply, demand and quality determinants of health intervention coverage. Enabling environmental determinants have been added to account for the social norms, policy, coordination and financial factors. As a general principle, the BNA approach contributes to, and complements the existing planning and monitoring structures [9, 10].

The aim of this work is to describe the application of the BNA process to assess gaps in immunization services at the regional level in Ghana and how it could guide planning and implementation of activities to sustain the gains in Immunization coverage. In addition, it describes baseline targets and indicators upon which progress in Immunization services in Ghana could be evaluated by 2018.

## Methods

A national assessment in May 2015 used both quantitative and qualitative approaches to assess gaps in delivery of immunization services. This was undertaken to assess barriers to coverage of services and included desk review, field visits and key informants interviews. This involved all ten regions in Ghana and two districts with the lowest coverage for the third pentavalent Immunization (penta-3).

***The Bottleneck Analysis Process for this gap analysis:*** involves the following critical steps:

i. Review performance of the selected programme, using the established core indicators
ii. Select the tracer interventions on basis of the reported performance. It should be an intervention with the lowest coverage, one that falls farthest from the national target or the one that requires most strengthening in order to achieve impact
iii. Identify the bottlenecks (obstacles or constraints within the process of service provision) and disparities (inequitable or non-uniform distribution or availability of services) in relation to the target population
iv. Conduct the causal analysis to determine the immediate, intermediate and structural causes of the bottlenecks and disparities
v. Identify the solutions/strategies that would be required to address the identified bottlenecks and disparities

### The BNA framework

**Fig. 1:**
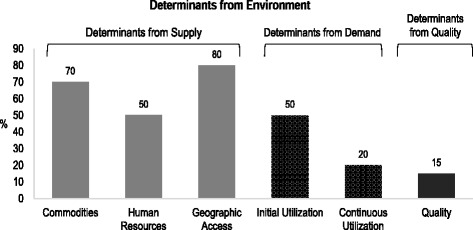
Graphical Presentation of the BNA Framework

The BNA framework is premised on the notion that effective coverage of services is influenced by four main factors or determinants namely: supply, demand, quality and environment. The BNA Tool produces a graphical output (Fig. 1) that facilitates identification of the key bottlenecks.

1. *Supply determinants* of services are predominantly controlled by the health care delivery system and have three important components: *commodities, human resources* and *geographic access.*
2. *Demand* determinants of services are predominantly controlled by the community, have two important components *initial utilisation* and *continuous utilisation* of services
3. *Quality* determinants of services are predominantly controlled by the health care delivery system and relate to the services being able to meet the *quality* standards set within national guidelines
4. *Environmental* determinants of services are cross-cutting factors that include the policy and regulatory frameworks, management, coordination and the socio-cultural as well as economic related factors

Identification of the low bars on the supply-determinant side of the graph as well as a sharp drop from one bar to the next on the demand and quality-determinant side of the graph enables managers to identify and prioritize bottlenecks to the effective health service coverage. Once strategies have been put in place to address the bottlenecks, changes in the bar graphs can be used to monitor the reduction of bottlenecks at periodic intervals.

#### Data collection

A Modified Bottleneck Analysis Tool (based on Microsoft excel 2013) was used as the data collection instrument for this national assessment. The Tool is based Microsoft Excel and generates simple graphs that display the six determinants of health service coverage on supply, demand and quality indicators (illustrated in Fig. 1) and thus facilitate identification of key bottlenecks that influence effective coverage. The section for data input is fully linked with the section for display of outputs. The BNA Tool is highly versatile and can be easily adopted for use at the national, regional, district as well as sub-district levels. The BNA Tool directly utilized data from District Health Information Management System (DHIMS), the Multi-Indicator Cluster Surveys [11], the Demographic and Health Survey (DHS) and programme monitoring and review reports in Ghana. Each region and district input their real service coverage data into the tool to generate the bars from which they identified the bottlenecks. EPI specific indicators used for assessing the supply, demand and quality determinants are shown in Table 1.

**Table 1:** Immunization specific indicators used for immunization gap assessment in Ghana

Determinant	Indicator	Definition of Indicator
Supply Side	Commodities	Proportion of health facilities without stock-outs of all vaccines and devices (0.5 ml & 005 ml Auto Destruct syringes) during the last year
	Human Resource	Proportion of health facilities with at least 80% of health care workers who have had in-service training on EPI (Routine Immunization) within last 2 years
	Geographic Access	Proportion of communities in region with a fixed or outreach EPI service delivery point
Demand side	Initial Utilization	Proportion of infants aged 0-11 months who received first pentavalent vaccine during the past year in region/district
	Continuous Utilization	Proportion of infants aged 0-11 months who received third pentavalent vaccine during the past year in region/district
Quality	Effective Coverage	Proportion of infants aged 0-11 months fully immunized (had all basic vaccines) during the past year in region/district

#### Outcome measures and data analysis

Outcome measures selected for the gap assessment were programme and service delivery indicators on routine EPI Immunization for infants (0-11 months), measles second dose 24-35 months and tetanol diphtheria toxoid Immunization for women (women in reproductive age 15-49 years and pregnant women). The base line year for this assessment was 2014 i.e. 2014 service delivery data were input into the BNA tool. National EPI coverage targets were used as the benchmark to assess gaps in regional and district service coverage indicators.

Outputs from the BNA tool by all regions and priority districts in Ghana were analyzed by simple descriptive statistics such as frequency, proportions and ratios. The principles of BNA were applied to identify bottlenecks and assess gaps using national level immunization coverage targets as the benchmark. Causality analysis were conducted to identify barriers resulting in these bottleneck and determining strategies to remove the bottlenecks to sustain immunization services in Ghana.

#### Ethical issues

All data used were aggregated data at regional and district level and had no link to individuals. In all cases, documentations and computerized records were kept secure and accessible to persons directly involved in developing the operational plans. The office of the Policy, Planning, Monitoring and Evaluation Division of the Ghana Health Service gave approval for documentation and dissemination of the BNA process in Ghana.

## Results

Ghana has made tremendous progress over the years in immunization service coverage and has increased the number of vaccines in the programme from six in 1978 to 12 in 2013 (as in Table 2).

**Table 2:** Introduction of different vaccines into the EPI in Ghana (EPI Annual Report, 2014)

Year of Introduction	Number of vaccines	Constituent vaccines
1978	6	*BCG, OPV, DPT, Measles
1992	7	Yellow Fever
2002	9	Pentavalent- DPT, Hepatitis B; Haemophilus influenza b
2012	11	Rotavirus vaccine; Pneumococcal vaccine
2013	12	MR- Measles, Rubella

BCG- Tuberculosis vaccine, OPV- Oral polio vaccine, DPT- Diphtheria, Pertussis and Tetanus vaccines, Measles

**Fig. 2:**
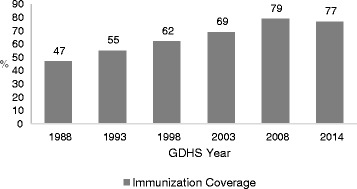
Trends in immunization coverage in Ghana

**Fig. 3:**
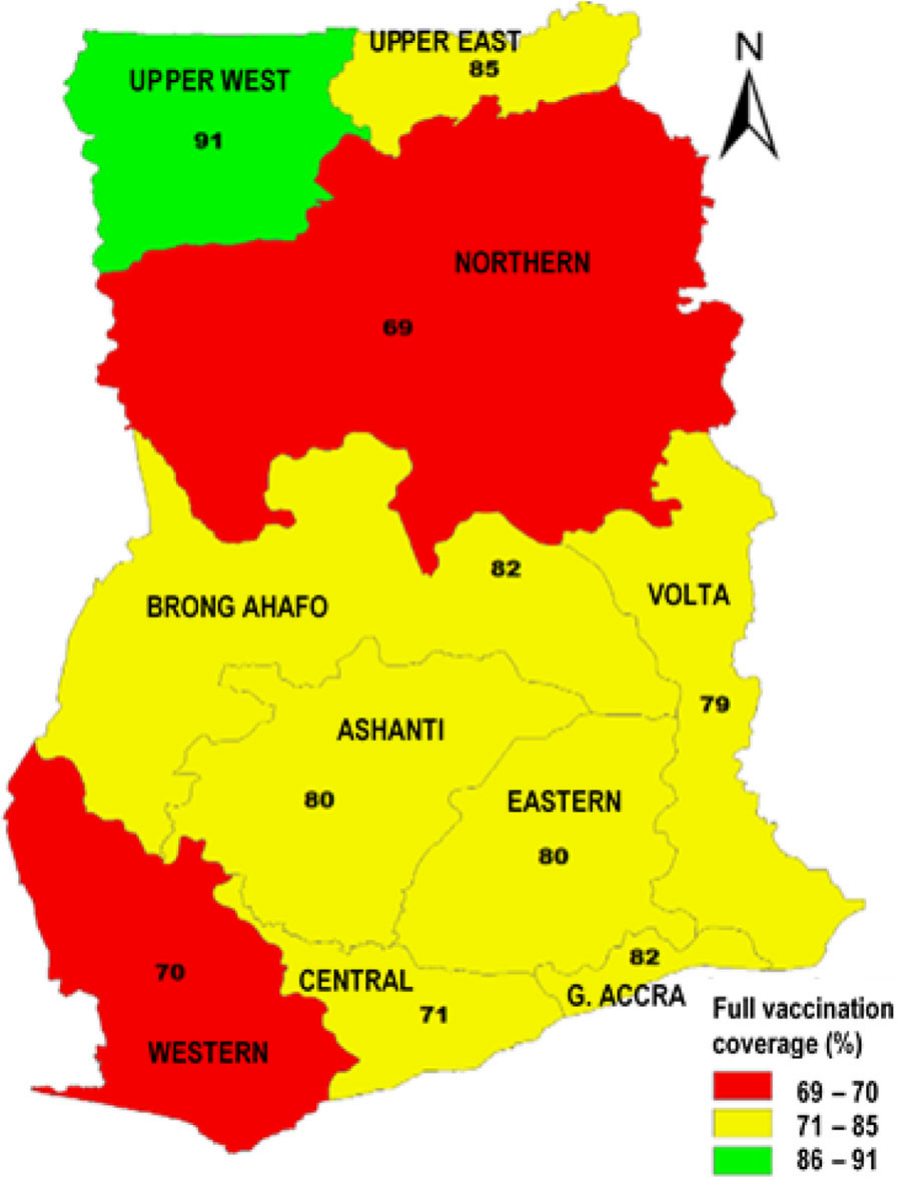
Trends in Fully Immunized Child per region in Ghana, 2014

Overall, the 2014 Ghana Demographic and Health Survey indicates that the fully vaccinated coverage rate for immunization for children in Ghana was 77% from a low of 47% in 1998, illustrated in Fig. 2 and national 3coverage for fully immunized child per region in Fig. 3.

### Key bottlenecks (gaps in coverage) for immunization services

**Fig. 4:**
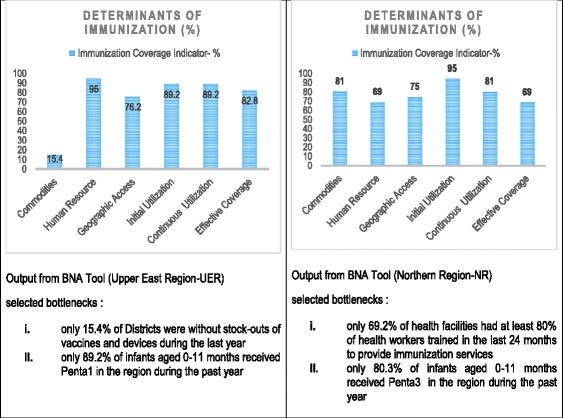
Examples of Bottleneck Identified at the regional level

All the regions and districts identified bottlenecks (gaps in coverage) from the tracer interventions based on the principles of BNA. Fig. 4 is a graphical output from the BNA Tool for Immunization by two regions and two districts regions.

Table 3 summarizes the gaps in immunization service coverage by all the ten regions in Ghana. The national target for all childhood immunization in Ghana is 95%. The BNA process apart from assessing the gaps enabled the regions to set realistic outputs in immunization coverage services by 2018.

**Table 3:** Regional disparities and gap analysis in immunisation services and expected outputs in sustaining national immunisation services in Ghana

Region	Gaps in coverage by end of 2014	Expected outputs by end of 2018
Ashanti	• First dose measles immunization (Measles 1) coverage of 78.9%	• Have 95% of infants aged 12-23 months fully immunized by 2018 (measles2)
	• Second dose measles immunization (Measles 2) coverage of 77%	• Have 80% facility staff trained on EPI
	• Fully immunized infants aged 0-11 months rate of 66. 9%	• Communities with outreach/static points increased from 63.9 to 80%
	• Only 63.9% of Communities have outreach/static points	
Brong Ahafo	• First dose measles immunization (Measles 1) coverage of 82.2%	• Children 0-11months vaccinated increased from 82.2 to 95%for measles 1
	• Second dose measles immunization (Measles 2) coverage of 75.1%	• Children 18-23months vaccinated increased from 75.1 to 95% for measles 2
	• Tetanol-diphtheria (Td2+) coverage of 73%	• Women with 2 or more tetanol-diphtheria vaccinations (Td2+) increased from 73 to 80%
Central	• Fully immunized infants aged 0-11 months rate of 70.9%	• Proportion of health facilities who had at least 80% of health care workers trained on EPI (Routine
	• Second dose measles immunization (Measles 2) coverage of 59.2%	Immunisation) within last 2 years increased from 35% in 2014 to 80%
	• Tetanol-diphtheria (Td2+) coverage of 60.8%	• Proportion of communities in the region which had a fixed or outreach EPI service delivery point increased from 55.5% in 2014 to 85%
	• Only 55.5% of Communities have outreach/static points	• Proportion of infants aged 0-11 months fully immunized increased from 70.9% in 2014 to 80%
	• Only 35% of health facilities had at least 80% of health care workers trained on EPI within last 2 years	• Proportion of children aged 12- 23 months who received second dose measles during the past year in region increased from 59.2 in 2014 to 80%
		• Proportion of expected pregnant women who received Tetanol-diphtheria (Td2+) during the past year in region increased from 60.8% in 2014 to 75%
Eastern	• Only 60% of health facilities had health care workers trained on EPI within last 2 years	• Eighty percent (80%) of health facilities have their staff receiving in-service training on EPI (Routine immunisation) in the region
	• Second dose measles immunization (Measles 2) coverage of 64.5%	• Coverage of children aged less than 23 months receiving second dose measles increased from 64.5 to 95%
	• Tetanol-diphtheria (Td2+) coverage of 31.2%	• Tetanol-diphtheria (Td2+) immunisation of expected Pregnant women increased from 31.2 to 60%
Greater Accra	• Only 66.7% of health facilities had health care workers trained on EPI within last 2 years	• Have 90% of facilities trained at least 80% of their Health Care Workers on routine EPI
	• Fully immunized infants aged 0-11 months rate of 70. 9%	• Have 80% of children aged 0-11month are fully immunized
Northern	• Fully immunized infants aged 0-11 months rate of 69%	• Health care workers trained in EPI increased from 69.2 to 95% in Northern region
	• Second dose measles immunization (Measles 2) coverage of 47%	• EPI service delivery points in Northern region increased from 75 to 95%
	• Tetanol-diphtheria (Td2+) coverage of 85%	• Children 0-11months have receiving third dose of pentavalent vaccine in Northern region increased from 80 to 90%
	• Only 69.2% of health facilities had health care workers trained on EPI within last 2 years	• Second dose measles coverage increased from 47 to 85% in Northern region
	• Only 80% of Children 0-11months had third dose of pentavalent vaccine (Penta3)	• Tetanol-diphtheria (Td2+) coverage increased from 85 to 90%
	• Only 75% of Communities have outreach/static points	• Coverage of fully immunized children increased from 69 to 80%
Upper East	• Only 2 of the 13 district were without stock outs of vaccines and logistics for immunization	• Second dose measles coverage increased from 43 to 60%
	• Second dose measles immunization (Measles 2) coverage of 43%	• Tetanol-diphtheria (Td2+) increased from 68.1 to 70%
	• Tetanol-diphtheria (Td2+) coverage of 61.8%	• Reduced number of districts with stock outs of vaccines and logistics at all levels
	• Only 58.3% of Communities have outreach/static points	• Improved access to immunisation services in the region
Upper West	• None of the health facilities had at least 80% of health care workers trained on EPI within last 2 years	• The number of health facilities with at least 80% of staff trained in EPI increased from 0 to 60%
	• Fully immunized infants aged 0-11 months rate of 91.2%	• The coverage of fully immunized infants age 0-11 months increased from 91.2 to 98%
	• First dose measles immunization (Measles 1) coverage of 76.0%	• The coverage of first dose measles immunization for infants 0-11 months increased from 76.0 to 90%
	• Second dose measles immunization (Measles 2) coverage of 70.1%	• The coverage of second dose measles immunization for children 12-23 months increased measles from 70.1 to 85%
Volta	• Only 69.8% of Communities have outreach/static points	• proportion of communities in region with a fixed or outreach EPI service delivery point increased from 69.8 to 80%
	• Fully immunized infants aged 0-11 months rate of 78.2%	• proportion of infants aged 0-11 months fully immunized during the past year in region increased from 78.2 to 90%
	• Second dose measles immunization (Measles 2) coverage of 64.2%	• proportion of children aged less than 23 months who received second dose measles immunization during the past year in region increased from 64.2 to 90%
	• Tetanol-diphtheria (Td2+) coverage of 55.8%	• proportion of expected pregnant women who received Tetanol-diphtheria (Td2+) during the past year in region increased from 55.8 to 90%
Western	• Only 53% of health facilities had health care workers trained on EPI within last 2 years	• Proportion of health facilities with at least 80% of health care workers trained on EPI (Routine Immunisation) increased from 53% in 2014 to 70%
	• Only 32.6% of Communities have outreach/static points	• Communities in region with a fixed or outreach EPI service delivery point increased from 32.6% in 2014 to 60%
	• Fully immunized infants aged 0-11 months rate of 65%	• Infants aged 0-11 months fully immunized increased from 65% in 2014 to 85%
	• Second dose measles immunization (Measles 2) coverage of 64.8%	• Children aged 12-23 months who had received second dose measles immunization (Measles 2) increased from 64.8% in 2014 to 85%
	• Tetanol-diphtheria (Td2+) coverage of 52.6%	

### National target for all childhood immunizations in Ghana is 95%

Based upon the gaps identified, regional and national immunization action plans were developed to prioritise interventions for correction of the identified bottlenecks and disparities by each region. The evidence based strategies proposed by the regions and districts were based on four thematic areas, Capacity Building, Procurement and logistics, Community Mobilization and Engagement with partners and stakeholders and Monitoring and coordination. Results are are summarized in Table 4.

**Table 4:** Summary of Key activities and actions resulting from the gap analysis on immunisation services in Ghana through the BNA approach

Strategic intervention	Key activities
Capacity Building	• Conduct EPI Training Needs Assessment in all the districts
	• Train regional , district and sub-district teams and health Staff (specific numbers and duration of training were provided by regions/districts) on the following:
	❖ Microplanning and mapping of existing static and outreach points
	❖ management and strategies to increase immunisation practice and coverages
	❖ logistics and cold chain management
	❖ Data management and reporting into national data system
	❖ EPI Coverage Survey
	❖ Identification and management of adverse events following immunisation (AEFI’s)
	❖ interpersonal communication and customer care
	• Strengthen service delivery within the second year of life to increase second dose of measles immunization at 18 months
	• Prioritize EPI activities and incorporate in the Integrated Regional Budget
	• Orient newly posted lower level and community health delivery staff (e.g. Community Health Officers) on EPI protocols
	• Build capacity of regional and district teams on proposal writing and grant applications to improve fund raising activities
Procurement and logistics	• Procure vehicles, motorbikes and boats and distribute according to need
	• Create centralized maintenance system for vehicles at regional and district levels
	• Conduct cold chain inventory
	• Procure and supply vaccine refrigerators, cold boxes, vaccine carriers & stabilizers to regions and district based on need
	• Provide adequate supplies of EPI tally books, vaccine ledgers, updated policies and field guide for use at services delivery points and facilities
	• Maintain quarterly distribution of vaccines and logistics from national to regional levels and monthly distribution of vaccines and logistics to districts to avert shortages
	• Maintain quarterly planned preventive maintenance of cold chain equipment throughout the year at the regional level
Community Mobilization and Engagement with partners and stakeholders	• Continuous education of women on the importance of card retention for Tetanol-diphtheria (Td2+) data monitoring
	• Improve communication at the community level through local radio/FM using messages and jingles in local languages
	• intensify health education at all service delivery points
	• conduct regular quarterly meetings with political and community leaders at district and sub-district levels
	• Hold regional stakeholders meetings to mobilize support
	• Regular community durbars, community advocacy meetings and radio discussions on EPI services
	• Conduct community surveys at the district to assess levels of satisfaction
	• Reactivate mother support groups in the communities
Monitoring and coordination	• Develop a composite monitoring and supervision plan at all levels
	• Conduct half yearly monitoring and supervisory visit from national to the 10 regions
	• Conduct quarterly monitoring and supervisory visit from region to districts
	• Conduct bi-monthly monitoring and supervisory visit from districts to sub-districts
	• Conduct annual EPI cluster survey
	• Review staff postings in all districts to ensure equity in health staff distribution by regional level
	• Conduct quarterly data validation meetings for district and sub-districts in the regions
	• Monitor and evaluate monthly outreach services for each district (planned versus conducted) by regional level
	• Develop areas for peer review and encourage peer review among districts by regional level
	• Submit plans for increasing vaccination sites by districts to region annually
	• Conduct quarterly EPI data quality and self-assessment at the district level
	• Organize half yearly coaching and mentoring visits to regions by national level

The gap analysis resulted in the development of a national immunization strategic plan in December 2015 and development of regional immunization operations plans and monitoring and evaluation framework for the national immunization strategic plan from 2016-2018.

### National effect of the immunization Gap assessment

The assessment identified major stakeholders in the health service arena who contribute to immunization services and who should be engaged continuously to sustain the gains in immunization coverage in Ghana. These were identified as Ministry of Health and Ghana Health Service; United Nations Organizations (UNICEF, WHO); other partners (Bilateral agencies, international and local NGOs); local Government agencies (Regional Coordinating Council, District/Municipal Assembly); the Media; and the community (Traditional, Religious and Opinion leaders).

In addition, the BNA assessment provided the basis for the development of national and regional immunization operational plans for 2016-2018.

## Discussion

Health inequities occur when health services are not accessible or utilized by certain people, based on their gender, socio-economic status, ethnicity, geographic residence, or other characteristic. These inequities can result from a lack of resources required to meet the needs of vulnerable populations [10]. Consequently, disadvantaged populations are at much higher risk of adverse health outcomes. In order to improve equity and ultimately reduce maternal, newborn, and child mortality, data-driven decision making and evidence-based planning is essential to channel limited health resources to the target population.

There are many national policies and guidelines for effective interventions to reduce child mortality, these interventions do not always reach those who need them most due to bottlenecks within and outside the health system [10]. Even for those with access, care may not be of good quality or be fully utilised in a manner appropriate to their needs [12]. Tanahashi’s 1978 work to clarify the concept of health services coverage and his approach to evaluate the effectiveness of coverage [9] forms the basis of UNICEF’s conceptual model and approach to conducting analyses of health system bottlenecks [10]. In 2010, UNICEF developed the Monitoring of Results for Equity System (MoRES) as part of its central focus on equity particularly for the most vulnerable groups [13]. MoRES aims to make more intensive and strategic use of data in order to inform equitable policy development, to mobilize stakeholders to identify and solve key bottlenecks to achieving equitable health outcomes, and to strengthen health system performance at all levels, particularly for vulnerable populations [13].

The bottleneck analysis approach (BNA) is used to identify barriers, bottlenecks and enabling factors that either constrain or advance the achievement of desired outcomes for vulnerable populations. It is based on the principle that certain conditions or determinants need to be fulfilled in order to achieve effective coverage of services, practices and systems. [9, 10]. BNA links data to evidence, and subsequently to actions, which is central to reducing barriers to service utilisation and promoting efficient resource use to improve children’s health and well-being [13]. The key elements of the system include an analytical structure that focuses on deprivation determinants, indicator identification to measure bottlenecks to reaching vulnerable populations and data collection and reporting on a frequent basis at sub-national levels for identified indicators. These determinants are key to developing pro-equity health systems and implementing effective programs with positive impacts [13].

The BNA was adopted analysis gaps in immunization services at the sun-national level in Ghana to sustain the gains made over the years. This approach deviated from the status-quo of planning at the national or central level which has hitherto been the case. It moved planning to the regions and districts employing real service coverage data to generate evidence of service delivery gaps. It enabled service providers to appreciate the usefulness of the data they generate and highlight clearly the deviations of their coverage indicator from that of the national, regional and district targets. Importantly, the adapted Tanahashi model, used with data disaggregated by geographic area, and other population attributes identified disparities in access and use of services among sub-national groups. Combined with a causal examination of non-financial and financial barriers to service use among at-risk populations, a more equity-focused set of health policies, strategies, and investments were subsequently be developed.

This gap analysis resulted in the development of individualized planning for each region and district, unlike in previous years where sub-national plans were very similar to each other since they were developed from a common template. It also garnered teamwork spirit and togetherness because teams now have to sit together and use various BNA tools to develop their plans as well as creating ownership by regions of their plans. This is because regions and districts used their own data to develop their plans unlike previous planning sessions where standardized templates were handed down for use.

The immunization implementation strategies developed with the BNA approach have been aligned to the national and regional EPI strategies, including strengthening of coordination mechanisms; increasing the demand through community involvement; improvement in the quality of EPI and Immunization; strengthening the implementation capacity of health facilities; and implementation of innovative approaches [14–16]. These are stemmed in the context of eight core components of routine Immunization, policy, standards, and guidelines; governance, organization, and management; human resources; vaccine, cold chain, and logistics management; service delivery; communication and community partnerships; data generation and use; and sustainable financing [17].

Above all, the gap analysis resulted in the development of a national immunization strategic plan in December 2015 and the development of regional immunization action plans, and monitoring and evaluation framework for the national immunization strategic plan from 2016-2018. The BNA approach will be used to assess how far identified gaps have been addressed in Ghana by 2018.

### Key lessons learned

Key lessons from the BNA approach in this gap analysis in Ghana concerned data and data quality. Good quality data is essential for effective planning and analysis, and data validation to improve data quality and accuracy is a core activity that should be undertaken by all the regions. Another important concern was the poor attitude of staff toward data capture and record keeping at all levels to achieve effective coverage of service indicators. This is critical because national or regional level data depends on data generated from the lowest level of service delivery; hence the need to put in measures to generate good quality data for performance assessment.

Based on the lessons learned, specific health system factors were identified for sustaining gains in immunization include, districts, sub-districts and community level systems being supported with basic logistics and equipment for vaccine storage to provide the full complement of immunization services. In addition, to overcome implementation challenges, regions and districts should encourage cooperation in the work function of sub-districts and community levels and should seek to strengthen the community based health planning and services (CHPS) in all districts and strengthen coordination function at all levels of the health system.

### Study limitation

The modified Microsoft Excel-based BNA tool used in Ghana for EPI assessment did not explicitly capture indicators on policy, legal, social norms and budget-related factors that shape the determinants of health service coverage. However, these cross-cutting factors were systematically considered as part of analyzing each identified bottleneck during the causal analysis. Incomplete data or low quality data for the base year 2014 in some regions and districts were a major challenge in identifying real bottlenecks to develop evidenced based plans. In addition, discussions of perceived health system barriers depended on knowledge and expertise of the health service provider and how well informed they were on health system issues regarding immunization.

## Conclusion

The BNA tool assist in the identification of gaps and health coverage challenges in immunization services in Ghana. Poor quality and incomplete data impact negatively on the gap assessment and subsequent development of micro plans and monitoring framework. The BNA tool is versatile enough to be applied to other health system interventions and for performance assessment in similar low income settings.

The assessment applying the BNA Tool provided baseline coverage indicators on immunization across the ten regions which resulted in the development of regional and national operational plans for immunization in Ghana. It will be the baseline for evaluating the national programme in three years.

## Abbreviations

BNA: Bottleneck analysis
CBV: Community based volunteers
CHOs: Community health officers
DDHS: District director of health services
DHIMS: District health information management System
DHMT: District health management team
GHS: Ghana health services
MDG: Millennium development goal
MOH: Ministry of health
MoRES: Monitoring of results for equity system
NGOs: Non-Governmental organization
TT/TD: Tetanus diphtheria immunization
UNICEF: United nations children fund
UWR: Upper west region
VPDs: Vaccine preventable diseases
WHO: World health organization
